# Exploring Seipin: From Biochemistry to Bioinformatics Predictions

**DOI:** 10.1155/2018/5207608

**Published:** 2018-09-19

**Authors:** Aquiles Sales Craveiro Sarmento, Lázaro Batista de Azevedo Medeiros, Lucymara Fassarella Agnez-Lima, Josivan Gomes Lima, Julliane Tamara Araújo de Melo Campos

**Affiliations:** ^1^Laboratório de Biologia Molecular e Genômica, Departamento de Biologia Celular e Genética, Centro de Biociências, Universidade Federal do Rio Grande do Norte, Natal, RN, Brazil; ^2^Departamento de Medicina Clínica, Hospital Universitário Onofre Lopes, Universidade Federal do Rio Grande do Norte, Natal, RN, Brazil

## Abstract

Seipin is a nonenzymatic protein encoded by the* BSCL2* gene. It is involved in lipodystrophy and seipinopathy diseases. Named in 2001, all seipin functions are still far from being understood. Therefore, we reviewed much of the research, trying to find a pattern that could explain commonly observed features of seipin expression disorders. Likewise, this review shows how this protein seems to have tissue-specific functions. In an integrative view, we conclude by proposing a theoretical model to explain how seipin might be involved in the triacylglycerol synthesis pathway.

## 1. Introduction

Lipodystrophies are rare diseases related to adipose tissue commitment [1]. For years, researchers pursued several candidate genes to associate and explain the biochemical mechanisms that underlie the clinical manifestations. The two pioneers of congenital lipodystrophy studies were Waldemar Berardinelli in 1954 [2], followed by Martin Seipin in 1963 [3]. Described as autosomal recessive diseases, different genes were tested as a candidate related to the physiopathology of lipodystrophies. In 2001, Magré et al. associated mutations in a specific locus of the 11q13 chromosome with type 2 Berardinelli-Seip congenital lipodystrophy (BSCL type 2) [4]. Therefore, they named the protein encoded from the* BSCL2 *gene as “seipin” as a tribute to Martin Seip. Because the molecular function of seipin was unknown, several investigators started to study its role in the biology of adipogenesis. Seventeen years later, seipin is still far away from being fully understood.

Seipin is a protein located in the endoplasmic reticulum (ER) membrane [5]. The* BSCL2* gene is highly expressed in the testis and some regions of the human brain, such as the spinal cord, frontal lobe cortex, and regions related to the regulation of energy balance, such as the hypothalamus and brainstem [4, 6, 7]. In mice, its expression is high in the motor and somatosensory cortex, mesencephalic nucleus, cranial motor nuclei, thalamic and hypothalamic nuclei, reticular formation of brainstem, and vestibular complex [8]. The human protein atlas databank (https://www.proteinatlas.org/ENSG00000168000-BSCL2/tissue) [9, 10] also confirms that the* BSCL2* gene is highly transcribed in the human brain. Besides, seipin is upregulated during* in vitro* hormone-induced adipogenesis [4, 11] and high expression in fully differentiated adipose tissue isolated from mice was also observed [12].

**Figure 1:**
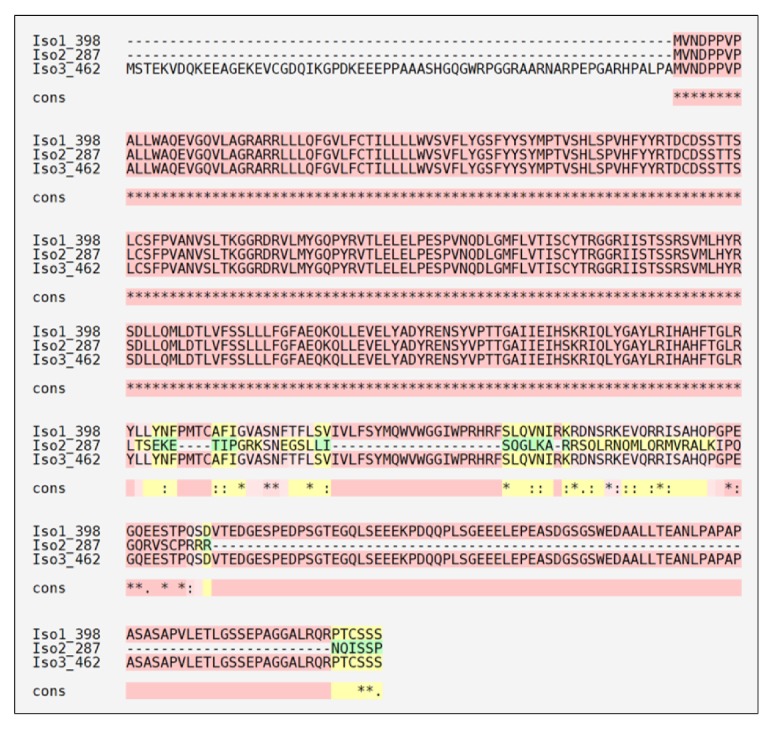
**Human seipin isoforms.** Multiple alignments of seipin isoforms were performed through T-Coffee [32]. Isoform 3 is the biggest with 462 amino acids, followed by 1 and 2 with 398 and 287 amino acids, respectively. UniProt [14] considers seipin isoform 1 as the canonical one. Pink color represents identical alignments; yellow corresponds to similar alignments; and green regions show different alignments. **∗** corresponds to an equal match and the differences are highlighted by . and **:** symbols. Cons: consensus sequence; Iso: isoform.

Two of the most famous primary bioinformatics databanks, NCBI [13] and UniProt [14], reveal three* BSCL2* transcription variants that produce three seipin isoforms. Seipin isoform 1 has 398 amino acids, while isoforms 2 and 3 have 287 and 462, respectively (Figure 1). Isoform 3 has a larger N terminus sequence, while isoform 2 is the most different from the other two, mainly at the C terminus site. After amino acids TGLR, only isoforms 1 and 3 are similar, even if the alignment tool tried to group isoform 2. Isoform 1 seems to be the most important seipin isoform and is considered the canonical one for the UniProt Site. Its sequence provides the nomenclature for more than 30 seipin mutations [4, 15–31]. Isoforms 1 and 3 are switched between these two databanks and some confusion might occur. However, we chose the nomenclature based on UniProt.

## 2. Seipin as an Oligomeric Transmembrane Protein

**Figure 2:**
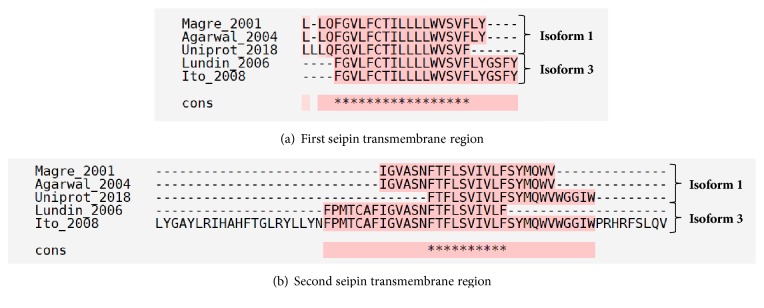
**Alignment of transmembrane regions of seipin.** Multiple alignments of seipin isoforms were performed through T-Coffee [32]. Many authors predicted the transmembrane regions of seipin and the amino acid positions are reviewed in Table 1 [4, 6, 14, 33, 34]. It is possible to observe that, even with differences, some regions are conserved in the prediction for the same isoform or between different isoforms. Isoform 2 was omitted due to the low number of works with it. Pink color represents identical alignments; yellow corresponds to similar alignments; and green regions show different alignments. *∗* corresponds to an equal match. Cons: consensus sequence.

In 2006, Lundin et al. predicted and experimentally confirmed that seipin is a transmembrane protein with two hydrophobic helices. They demonstrated its C and N terminus facing cytosol and concluded that seipin has a core/looping region inside the ER lumen. At the time, Lundin considered seipin with 462 amino acids and made some bioinformatics predictions with that isoform [33]. Many authors confirmed seipin regions localized through the ER membrane, although there are some differences in amino acids positions among the predictions (Table 1 and Figure 2) [4, 6, 33, 34].

**Figure 3:**
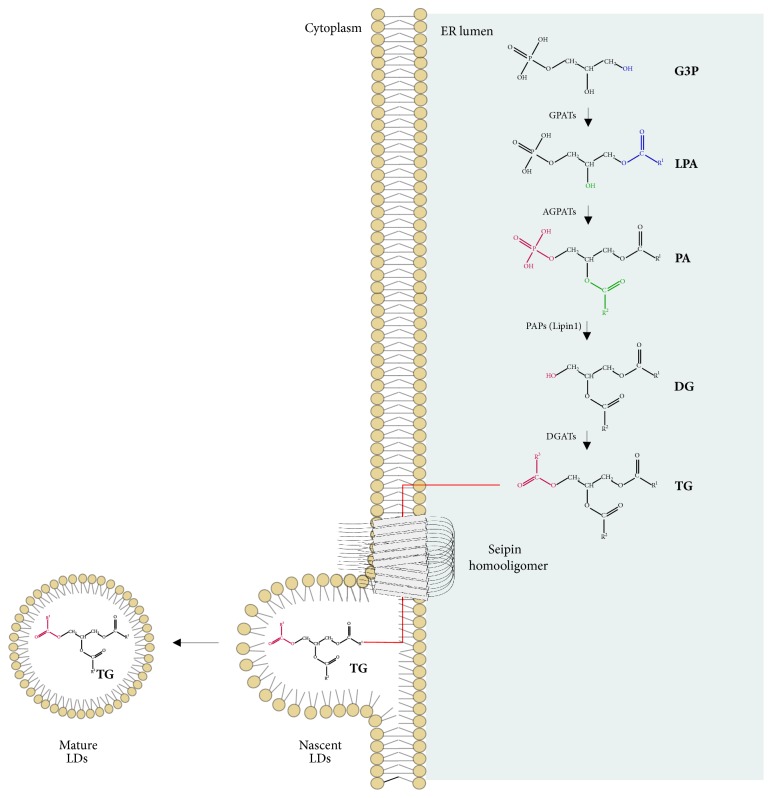
**Triacylglycerol synthesis and usual seipin localization.** During triacylglycerol synthesis, glycerol-3-phosphate acyltransferases (GPATs) catalyze the acylation at sn-1 position of glycerol-3-phosphate (G3P) and origin lysophosphatidic acid (LPA). Then, 1-acyl-sn-glycerol-3-phosphate acyltransferases (AGPATs) catalyze the acylation at sn-2 of LPA and give rise to phosphatidic acid (PA). Later, phosphatidate phosphatases (PAPs), as lipin1, can remove the phosphate group from PA and produce diacylglycerol (DG). Finally, diacylglycerol o-acyltransferases (DGATs) catalyze the acylation at the sn-3 position and give rise to triacylglycerol (TG) [35, 36]. In the same context, seipin comes as an oligomeric endoplasmic reticulum (ER) transmembrane protein that acts in lipid droplet (LD) assembly. ER and LDs were found to be neighbors, and seipin is concentrated in the communication regions between them, enabling the transfer of lipids recently synthetized to nascent LDs [37–39]. Pieces of the illustrations are from the SMART website [40].

In 2010, Binns et al. found that* Saccharomyces cerevisiae* seipin is a large protein complex. They suggested a stable homooligomer model of about nine subunits with a radially symmetric shape. For the authors, that structure resembled a toroid and appeared to be involved in the lipid droplet (LD) assembly organization [49]. Sim et al. confirmed seipin homooligomerization in human cell culture but found 12 subunits in a circular configuration [50]. The topology of seipin lacks evidence for any enzymatic domains or activity and some authors suggest that it may act as a scaffold for other proteins or play a structural role in membranes [51] (Figure 3).

Seipin, as an ER protein, affects the homeostasis of this organelle directly or indirectly in a tissue-specific way. The ER is a tubular organization specialized in the synthesis, mobility, and transport of proteins in eukaryotic cells. The tertiary structure of these macromolecules is essential for cell survival, and their accumulation out of the native conformations can compromise proteostasis [52]. As we will see in this review, seipin might be one of the proteins that can have a compromised folding and elicit an intracellular phenomenon called ER stress. Poorly folded proteins are able to bind chaperones and elicit the unfolded protein response (UPR). UPR is a marker of ER stress characterized by sequential reactions that may culminate in stress adaptation with protein ubiquitination and proteasome degradation. However, in unsolved stress situations, autophagy or apoptosis may arise [52–55].

## 3. Lipid Metabolism, Adipogenesis, and Lipid Droplets

Lipid anabolism consists of some reactions including the ones which synthesize fatty acids (FA) and triacylglycerol (TG). TG synthesis depends on FA availability and occurs in most cells, but mainly in adipocytes and hepatocytes (Figure 3). Cells usually receive FA from lipoproteins of blood in a fed situation and can use that lipid for energy production or TG synthesis for storage into LDs [56, 57]. Lipid droplets are born from ER and are present in almost all eukaryotic cell types. LDs work in lipid metabolism and energy production as the intracellular “house” of some neutral lipids, such as TG and cholesterol esters [58]. Small amounts of lipids may exist in the aqueous ambient of ER but, as their number increases during TG synthesis, LDs may bud on a monolayer surface. When they sufficiently grow towards the cytosol, they might become independent organelles [59], as shown in Figure 3.

Essentially, all cells have the potential to store TGs into LDs. Nevertheless, around 90% of these lipids are inside LDs in white adipose tissue (WAT). One of the first steps of TG catabolism is the lipolysis: the reactions that turn TG into glycerol and FA and that mostly happen in WAT. Indeed, this is the unique tissue able to supply FA to other tissues. Glucagon is a positive regulator of WAT lipolysis, allowing release and transport of FA to muscles, liver, and other tissues. There, oxidative steps of lipid catabolism can occur to produce energy [60].

While lipolysis and TG synthesis should happen mostly in fully differentiated WAT, the former is usually “silenced” during adipogenesis [45, 61]. Adipocyte differentiation starts with mesenchymal stem cells (MSCs) that produce the preadipocytes in the first step. Next, the preadipocytes turn into fully differentiated adipocytes in the second step. During adipogenesis, the protein peroxisome proliferator-activated receptor gamma (PPAR*γ*) is “the master regulator,” acting as a transcription factor. PPAR*γ* has its activity regulated in a tissue-specific manner, through coactivators and corepressors. Secondly, some transcription factors, such as CCAAT/enhancer-binding protein beta (C/EBP*β*) and cyclic AMP-responsive element-binding protein 1 (CREB1), are also important [62, 63].

## 4. Seipin-Related Diseases: Lipodystrophy and Seipinopathy

All disorders involving seipin are characterized by some nervous tissue commitment. It is interesting to note how the same protein is involved in distinct clinical manifestations: Berardinelli-Seip congenital lipodystrophy type 2 (BSCL type 2) and seipinopathies. BSCL type 2, classified as one of the most dangerous of lipodystrophies, is a recessive disease caused by loss-of-function mutations, characterized by a severe adipose tissue disorder that might affect cognition-related nervous tissue regions [20, 64, 65]. “Seipinopathies,” a term created to refer to specific motor neuropathies, are dominant diseases caused by gain-of-function mutations, mostly related to nervous tissue disorders: Silver syndrome (SS) and distal hereditary motor neuropathy (dHMN) [6].

Seipin study not only is crucial for rare conditions such as BSCL type 2 and seipinopathies but also might be important in obesity pathogenesis or treatment, because of the relationship of that protein with adipose tissue homeostasis. Next, we will discuss some seipin studies to understand the biochemistry of how that protein can affect lipid metabolism and be the central player of these diseases. We chose to divide the sections into adipogenic and nonadipogenic cells inspired by the paper of Yang et al., who proposed and proved different seipin functions for these two models [61].

## 5. Seipin Loss-of-Function

### 5.1. Seipin Loss-of-Function through Silencing or Knockout in Adipogenic Models

**Figure 4:**
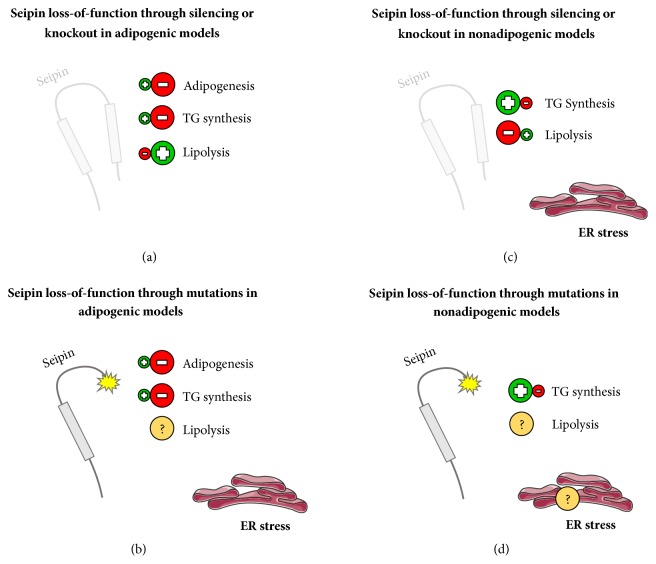
**Seipin loss-of-function.** We are proposing 4 different general models that usually happen with frequency under seipin loss-of-function. (a) Seipin loss-of-function through silencing or knockout in adipogenic models. Adipocyte maturation and TG synthesis are impaired, and ER stress was not found. (b) Seipin loss-of-function through mutations in adipogenic models. Adipocyte maturation and TG synthesis are impaired, ER stress was positively found, and there is a lack of information about the lipolysis situation. (c) Seipin loss-of-function through silencing or knockout in nonadipogenic models. TG synthesis was increased, lipolysis is impaired, and ER stress was positively found; (d) seipin loss-of-function through mutations in nonadipogenic models. TG synthesis was increased, and there is a lack of information about ER stress and lipolysis. (b) and (d) are the most representative situations of Berardinelli-Seip congenital lipodystrophy (BSCL) type 2. Positive symbols represent a process that is usually increased, while the negative symbols represent the opposite. The interrogation symbol represents a process that needs to be studied further. Pieces of the illustrations are from the SMART website [40].

Situations that impair* BSCL2* gene expression from a quantitative point of view are associated with problems in adipocyte maturation. This is strictly related to TG synthesis reduction and lipolysis stimulation (Figure 4(a)). 3T3-L1 mouse preadipocyte stem cells stimulated to differentiation in a* BSCL2* knockdown condition were associated with downregulation of genes responsible for TG accumulation. Additionally, the same group performed TG quantification by a direct measurement technique and showed lower TG content when compared with controls [11]. Other independent researches confirmed the association of these cells with reduced total lipid accumulation and content [61]. In other models, such as* BSCL2* knockdown murine embryonic fibroblasts (MEFs) stimulated to differentiation, decreased TG accumulation, and adipocyte maturation impairment, were found. Interestingly, that same group observed an increase in protein kinase A- (PKA-) activated lipolysis. Likewise, adipogenesis was restored with lipolysis inhibition [45]. TG synthesis impairment was also associated with downregulation of two important enzymes of this pathway: 1-acyl-sn-glycerol-3-phosphate acyltransferase 2 (*AGPAT2*) and the phosphatidate phosphatase* LPIN1 *(lipin1) in* BSCL2* knockdown during adipogenesis [11, 45].

Many studies have tried to explain why adipogenesis impairment is related to seipin loss-of-function. Researchers observed that induction of* BSCL2* transcription is an important event for the adipocyte differentiation and expression of some important adipogenic factors [12]. Chen et al. determined that* BSCL2* expression is only essential during the second phase of adipogenesis: differentiation of preadipocytes to fully differentiated adipocytes.* BSCL2* mRNA interference during that phase caused lower* PPARG* gene expression and adipogenesis impairment. However, treatment with a PPAR*γ* agonist rescued the process, proving that seipin is important for adipogenesis cascade at an upstream point compared with PPAR*γ* [11]. Another independent study also observed* PPARG* downregulation during mouse adipogenesis in a* BSCL2* knockdown situation [61]. The ER stress is dangerous to adipogenesis because of the suppression of PPAR*γ* expression [66]. Nonetheless, the research failed to associate* BSCL2* gene knockdown, adipogenesis, and ER stress [45].

### 5.2. Seipin Loss-of-Function through Mutations in Adipogenic Models

Situations that impair* BSCL2* gene expression from a qualitative point of view are also related to problems in adipocyte maturation. In the same way, this is strictly related to a decrease in TG synthesis (Figure 4(b)). Researchers observed a reduction in LD formation, associated with impaired lipid accumulation capacity during adipogenic differentiation of fibroblasts in patients with mutations E189X and R275X in seipin [67]. Moreover, other studies showed a reduction of almost 50% in TG content during differentiation of 3T3-L1 mouse preadipocyte stem cells carrying the A212P mutation. That group also observed downregulation of lipogenic genes and PPAR*γ*. As expected, adipogenesis was partially restored by agonists of that protein [68].

The most discussed clinical disease associated with seipin loss-of-function disorders is type 2 Berardinelli-Seip congenital lipodystrophy (BSCL type 2). The main characteristics of this lipodystrophy are the almost complete absence of adipose tissue together with mild to severe intellectual impairment. This condition is one of the most dangerous among human lipodystrophies and is associated with some cases of hypertrophic cardiomyopathy and secondary mitochondrial dysfunction [20, 25, 69]. The loss of body fat is also one of the most significant features, affecting both mechanically or metabolically active adipose tissue [70].

Regarding ER stress, 3T3-L1 mouse preadipocyte stem cells presenting the A212P mutation in the seipin gene and stimulated to adipocyte differentiation showed an increase in UPR [68]. Interestingly, this mutation was shown to change seipin localization in more than one study [12, 50], indicating that seipin mutations might activate UPR even if the protein is not in ER. Regarding adipocytes, independent authors observed that ER stress attenuates adipogenesis through repression of PPAR*γ* [66]. However, other research showed that seipin mutations that increase ER stress without compromising seipin function (N88S) are not enough to impair adipogenesis [68]. In such a way, seipin loss-of-function seems to be more important to adipogenesis commitment than possible ER stress.

### 5.3. Seipin Loss-of-Function through Silencing or Knockout in Nonadipogenic Models

Situations that impair* BSCL2* gene expression from a quantitative point of view were also studied in other cells without adipogenic stimulation. This is strictly related to stimulation of TG synthesis and a decrease in lipolysis (Figure 4(c)). Yeast model with deletion of Fld1, a human seipin homologue, shows an increase in TG synthesis and formation of supersized (giant) LDs [37, 38]. Human HeLa cells with* BSCL2* mRNA silenced also showed similar results, and overexpression of wild-type seipin reversed this phenotype. Additionally, the same group showed that* BSCL2* mRNA silencing in 3T3-L1 mouse preadipocyte stem cells positively regulates TG synthesis. However, they also observed small and clustered LDs, different from the yeast model [71]. In the same way, a* Drosophila* salivary gland model with* BSCL2* gene deletion presented ectopic LD formation and increased TG synthesis [72].

Mouse* BSCL2* gene deletion in hepatocytes showed LDs increased in number and size, as well as expression of genes implicated in their formation and stability. Stearoyl-CoA desaturase-1 (SCD1) acts in FA synthesis and seems to be negatively regulated in the presence of wild-type* BSCL2 *in hepatocytes. Hence, SCD1 knockdown reversed the phenotype associated with seipin deficiency. For the authors,* BSCL2* knockdown induces SCD1 expression and activity, leading to TG synthesis stimulation, lipolysis reduction, and LD expansion, at least in hepatocytes [73].

Liu et al. studied the effect of* BSCL2* gene deletion in fully differentiated mouse adipose tissue and found a progressive lipodystrophy. As expected, signals of TG accumulation were observed together with lipolysis impairment [74]. Similarly, Chen et al. observed increased lipid uptake gene expression, stimulation of TG synthesis, and FA synthesis in mouse residual epidermal WAT with* BSCL2 *gene deletion [75], which means that fully differentiated WAT seems to manifest characteristics of nonadipogenic cells during seipin expression disorders. The inclusion of fully differentiated adipocytes together with nonadipogenic cells might be unusual, but in terms of lipolysis and TG synthesis, these cells share similarities with nonadipogenic models. Indeed, both are not going through adipogenesis.

Specifically in mice adipocyte,* BSCL2* deletion was also associated with p38-mitogen-activated protein kinase- (MAPK-) dependent apoptosis increase. The authors observed that fibroblast growth factor 21 (FGF21) improved the consequences of seipin loss-of-function, through inhibition of p38-MAPK activity. This led to increased adiponectin plasma levels and metabolic homeostasis improvement [76]. Interestingly, mouse brown adipose tissue (BAT) functions do not seem to be affected much by* BSCL2* gene deletion when compared with WAT [77]. Indeed, deletion of seipin during mouse BAT adipogenesis is not sufficient to impair the whole process [78]. In spite of this, mouse fully differentiated BAT with* BSCL2* gene deletion displayed altered thermogenic capacity and some insulin resistance [79].

Seipin function is also important for assembly and homeostasis of LDs. In the human epidermoid carcinoma human cell line model (A431),* BSCL2* knockout triggered an aberrant LD morphology. The authors observed that seipin is enriched at the point of contact between ER and LDs, as shown in Figure 3, and the mutant seipin is not in that location anymore. For them, ER-LD contacts are morphologically abnormal in cells without seipin and this protein is also important for the stabilization of the contact between these two organelles, facilitating LD growth [80]. In the same year, another group showed similar results, proposing that seipin is required at the moment of transition between nascent to mature LDs, enabling lipid transfer to the nascent LDs in ER-LD contact sites [39].

Seipin is also highly expressed in testis. Testicular tissue from mice with* BSCL2* gene deletion also showed signals of TG synthesis stimulation such as a phosphatidic acid (PA) increase compared with controls. The authors discussed that lipid metabolism might be essential for testicular homeostasis and showed that mutations in seipin are also related to altered spermatozoid morphology, a phenomenon called teratozoospermia [81]. Indeed, further works with mouse* BSCL2* gene deletion observed male infertility with the absence of spermatozoids (azoospermia) and concluded that seipin presence is important for the late phase of spermatogenesis [77].

In neuron-specific seipin-knockout mice, Zhou et al. also observed downregulation of* PPARG*. It means that the PPAR*γ*-seipin relation is not only restricted to adipogenesis or adipose tissue [82]. Further work showed that* BSCL2* knockout mice could impair proliferation and differentiation of hippocampal cells through reduced PPAR*γ* levels. Indeed, this was a phenomenon reversed by PPAR*γ* agonists [83]. Similar results were recently obtained by a group who showed that seipin deficiency is related to lower expression of *α*-amino-3-hydroxy-5-methyl-4-isoxazolepropionic acid (AMPA) receptor through reduced ERK-CREB activities. Together, this phenomenon was reversed by PPAR*γ* activation [84]. Other studies also showed a reduced PPAR*γ* level in mouse neuronal* BSCL2* knockout. These mice had impaired adaptation to amyloid neurotoxins when compared with wild-type seipin mice. Together, the authors observed that neuroinflammation could be intensified in absence of seipin, through PPAR*γ* reduction [85]. Recently, Wang et al. showed that seipin deficiency in dopaminergic neurons enhances phosphorylation of *α*-synuclein and induces inflammation in an ER stress-independent manner. This phenomenon can trigger loss of dopaminergic neurons and is related to a decrease and increase in PPAR*γ* and glycogen synthase kinase-3 beta (GSK3*β*) activities, respectively [86].

Zhou et al. and Wang et al. did not find evidence of ER stress in neuron-specific seipin-knockout mice, respectively [82, 86]. In contrast, Liu et al. observed ER stress markers positively induced in adipose-specific seipin-knockout mice [74]. Zowalaty et al. also showed ER stress and increased apoptosis in mammary gland alveolar epithelial cells with* BSCL2* gene deletion and revealed the importance of seipin for lactation [87].* BSCL2* knockout was also associated with intensified cerebral ER stress after a transient middle cerebral artery occlusion situation in another study [88]. Moreover,* BSCL2* gene deletion in hepatocytes in mice also showed an increase of ER stress markers [73]. Interestingly, in a yeast model, ER stress was shown to stimulate LD formation and TG synthesis [89]. Therefore, this is a phenomenon that deserves more study in situations of seipin loss-of-function, as not all of the mutant seipin are able to accumulate in the ER and activate UPR [6].

### 5.4. Seipin Loss-of-Function through Mutations in Nonadipogenic Models

Situations that impair* BSCL2* gene expression from a qualitative perspective were also studied in other cells without adipogenic conditions. This is strictly related to TG synthesis stimulation (Figure 4(d)). Szymanski et al. studied a human fibroblast line obtained from a patient with a* BSCL2* nonsense mutation. Compared with the control, these cells presented many smaller and aberrant LDs [38]. In another study using* in vivo *seipin E253X mutation, the researchers also observed teratozoospermia and enlarged ectopic LDs in testis [81].

A study with some seipin mutations, such as A212P + S64R, Y106C, Y225L, and V99L, in human Epstein Barr virus-transformed lymphocytes showed more numerous and smaller LDs when compared with controls. Surprisingly, this might not be associated with increased TG synthesis, as the authors found TG content reduction in these cells when compared to the controls. However, the authors quantified TG after an overnight deprivation of serum in culture medium [90]. We hypothesize that this is a different situation compared with the* in vivo* constant hypertriglyceridemia, commonly found in BSCL type 2 patients [1]. Yet, these mutations do not significantly reduce seipin function as an interference mRNA (Figure 4(c)). A212P mutation, for example, does not seem to affect seipin quantitative expression but alters its localization [12, 50]. That substitution also does not affect the binding of seipin and lipin1 [50], a phenomenon that will be discussed further. Besides, that mutation could not inhibit LD formation in 3T3-L1 mouse preadipocyte stem cells without stimulation to differentiation [61].

Researchers found that hepatic steatosis is a common characteristic of people who inherited seipin loss-of-function mutations [1]. As we reviewed, previous works showed an increase in liver TG content in mice with* BSCL2* gene deletion when compared with controls [45, 91–93]. Nonetheless, lipodystrophy secondary effects have some importance for these results. Some authors observed that the specific seipin deficiency in mouse adipose tissue is mainly responsible for dyslipidemia, lipodystrophy, hepatic steatosis, and insulin resistance [94]. In such a way, metabolic dysfunction should not be expected in nonadipocyte-specific seipin deficiency. This was exactly the same result observed by Chen et al. in a mouse liver-specific deletion of the* BSCL2* gene, as these animals did not develop hepatic steatosis, proving that* BSCL2* is not autonomous to liver lipid homeostasis [93]. Similar results were also achieved by Wang et al. [95]. Researchers also observed that specific seipin deficiency in developing adipose tissue from mice is mainly responsible for lipodystrophy but not for severe hepatic steatosis, glucose intolerance, and insulin resistance [96]. Hence, we believe that BSCL type 2 metabolic features in nonadipogenic cells are caused by either secondary lipodystrophy-associated dysfunctions or some tissue-specific seipin loss-of-function. It is not correct to think that a phenotype, such as hepatic steatosis, is exclusively caused by seipin loss-of-function, without considering that patients' hepatocytes are constantly exposed to a high triacylglycerol blood content, for example.

## 6. Seipin Gain-of-Function

### 6.1. Seipin Gain-of-Function through Overexpression in Adipogenic Models

**Figure 5:**
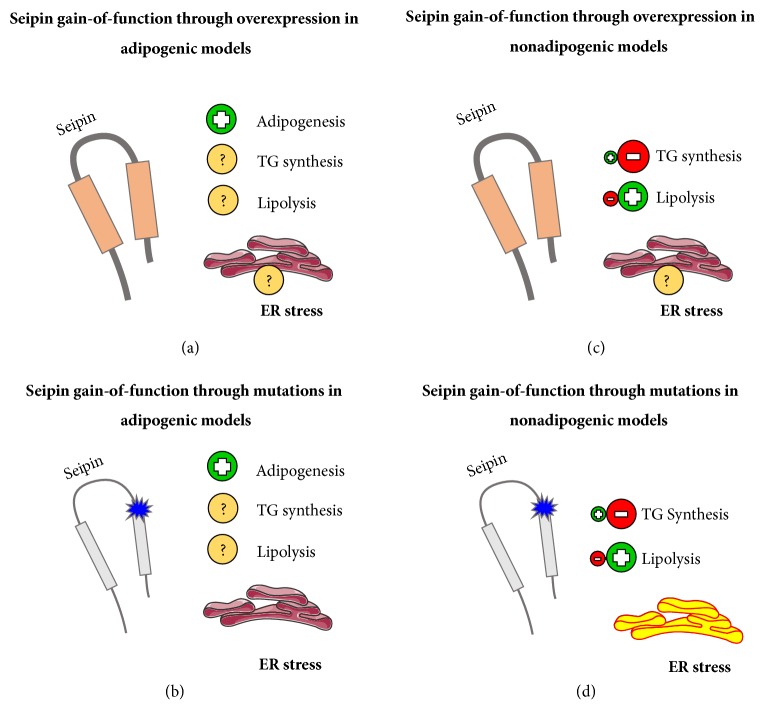
**Seipin gain-of-function. **We are proposing 4 different general models that usually happen with frequency under seipin gain-of-function. (a) Seipin gain-of-function through overexpression in adipogenic models. Adipocyte maturation was not impaired, but other parameters are still not clear. (b) Seipin gain-of-function through mutations in adipogenic models. Adipocyte maturation was not impaired even with ER stress. (c) Seipin gain-of-function through overexpression in nonadipogenic models. There was TG synthesis decrease, with lipolysis increase. (d) Seipin gain-of-function through mutations in nonadipogenic models. TG synthesis impairment was observed together with lipolysis increase and strong and significant ER stress. (b) and (d) models are related to seipinopathy. Positive symbols represent a process that is usually increased, while the negative symbols represent the opposite. The interrogation symbol represents a process that needs to be studied further. Pieces of the illustrations are from the SMART website [40].

To our knowledge, seipin wild-type quantitative overexpression during adipogenesis was studied only in a few works (Figure 5(a)), and Yang et al. observed that the preadipocyte reaches the differentiation in these conditions [61].

### 6.2. Seipin Gain-of-Function through Mutations in Adipogenic Models

Mutations that modify* BSCL2* gene expression from a qualitative perspective and give seipin a gain-of-function are not associated with problems in adipocyte maturation (Figure 5(b)). As expected, 3T3-L1 mouse preadipocyte stem cells stimulated to differentiate in the presence of N88S and S90L seipin gain-of-function mutations did not show defective adipogenesis. Besides, the authors observed that the presence of ER stress secondary to these mutations is not enough to cause adipogenesis impairment [68].

### 6.3. Seipin Gain-of-Function through Overexpression in Nonadipogenic Models

Situations that modify* BSCL2* gene expression from a qualitative perspective and give seipin a gain-of-function were studied in other cells without adipogenesis stimulation. This was strictly related to a decrease in TG synthesis (Figure 5(c)). Overexpression of wild-type seipin reduced TG and LD biosynthesis in both human HeLa and NIH3T3 cells [71]. Overexpression of seipin lacking its C terminus sequence or wild-type seipin dramatically reduced LD formation in hepatocytes AML-12 and 3T3-L1 mouse preadipocyte stem cells without differentiation stimulus [61].

The fully differentiated adipocytes have similar behaviors compared to other cells that are not in adipogenesis. Cui et al. overexpressed the human seipin exclusively in the mature adipocytes of mice. They observed a decrease in adipose tissue mass and lipolysis increase. Together, they concluded that seipin inhibits the lipid storage in mature adipocytes [97]. Here, we would like to invite you to compare Figure 4(c) with Figure 5(c). Even in opposite models, they point to a common finding that was concluded by Cui et al. [97]: in nonadipogenic cells (including mature adipocytes), seipin acts to limit lipid storage through TG synthesis impairment and to favor the lipolysis.

### 6.4. Seipin Gain-of-Function through Mutations in Nonadipogenic Models

Situations that modify* BSCL2* gene expression from a qualitative perspective and give seipin a gain-of-function are not associated with problems in adipocyte maturation and lipodystrophy. However, they give rise to the following seipinopathies (Figure 5(d)). SS and dHMN are two motor neuropathies that affect distal limb muscles. Individuals with SS have atrophy of the hands as the most marked manifestation and have mild to severe spasticity of the lower limbs, indicating involvement of upper motor neurons as well. Dominant mutations that compromise the N-glycosylation motif of seipin, such as N88S and S90L, are one of the genetic causes of SS and dHMN. These substitutions can form protein aggregates, a mechanism shared with some neurodegenerative disorders [5, 98, 99]. In a* BSCL2* knockout* Drosophila* model, the S90L mutation rescued the fat body, proving that it is gain-of-function. [72].

**Figure 6:**
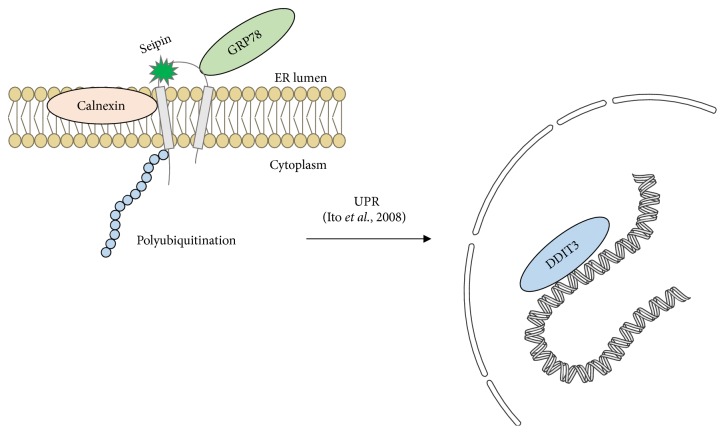
**Gain-of-function mutations in seipin elicit ER stress in seipinopathies. **Ito et al. proposed a model in which N88S and S90L mutations are able to disturb the seipin glycosylation site and generate ER stress and the unfolded protein response (UPR) [6]. They observed increased apoptosis as a result of the process. DDIT3 (also called CHOP) is a transcription factor responsible for the positive regulation of proapoptotic genes in response to ER stress. Calnexin (*CANX*) and GRP78 (also called BIP) are chaperones that work in UPR. As we reviewed, ER stress is not a phenomenon exclusively related to seipinopathy and might also be important for lipodystrophies. Pieces of the illustrations are from the SMART website [40].

Ito et al. studied mouse brain neuroblastoma (N2a) cells expressing N88S and S90L seipin mutations. In these cases, they observed a strong interaction between seipin and the chaperone calnexin (*CANX*), responsible for the correct folding of new ER-synthetized proteins, compared to controls. They suggested that seipin mutations were able to strongly activate the UPR and suggested that the unfolded seipin would physically attract these kinds of chaperones. In addition, ER stress seemed to be so intense that it triggered apoptosis. For them, neurodegeneration observed in motor neuropathies could be explained by the strong ER stress consequences [100]. Moreover, an* in vivo* model with transgenic mice expressing N88S seipin also confirmed ER stress as a significant phenomenon associated with seipinopathy [101]. That mutant seipin also localized in the ER, suggesting evidence of the presence of an unfolded mutated protein as a cause of ER stress [6, 100]. The mechanism illustrating ER stress in seipinopathy is shown in Figure 6.

Autophagy can be one of the many ER stress consequences. Indeed, mutant seipin in seipinopathy is also associated with the activation of that pathway in a mouse model [102], human embryonic kidney 293 cells (HEK293), and human non-small cell lung carcinoma cell line (H1299) [48]. Likewise, the expression of mutated seipin caused small diffuse LDs to fuse into larger ones and autophagy inhibition could reverse it. Not surprisingly, when the ER stress stimulator tunicamycin was added to the normal cells, it mimicked mutated seipin behavior. For them, autophagy is activated as an adaptive response to engulf and break down the LDs [48]. Other researchers manipulated astrocytoma and motor neurons to express N88S seipin and observed positive markers for ER stress but also a TG content diminution. As expected, supplementation with oleic acid and lipolysis inhibition were able to reverse these phenotypes. The authors proposed that lipolysis inhibition and new LD formation are associated with good prognosis of seipinopathy [103].

## 7. Merging of Genetic Concepts

**Figure 7:**
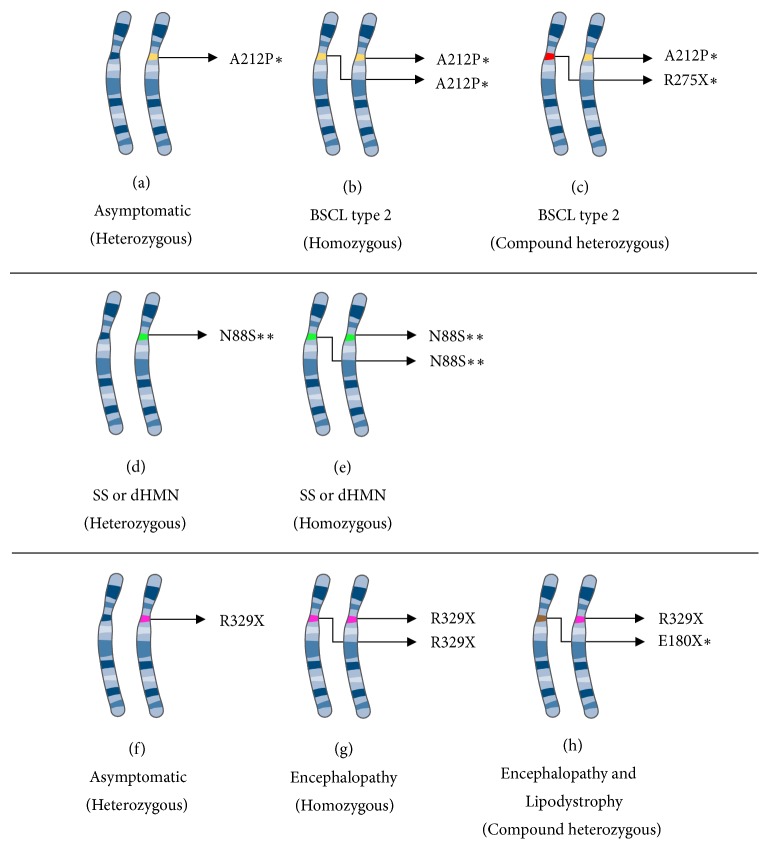
**Inheritance pattern of seipin-related diseases.** Here, we summarize the inheritance mechanism of seipin-related diseases. (a–c) BSCL type 2, a loss-of-function, and recessive disease. (d-e) Seipinopathies, gain-of-function, and dominant diseases. (f–h) Progressive encephalopathy with or without lipodystrophy (PELD) and a gain-of-function and recessive disease. Pieces of the illustrations are from the SMART website [40]. *∗* Or other mutations related to BSCL type 2. *∗∗* Or other mutations related to seipinopathy. BSCL: Berardinelli-Seip congenital lipodystrophy; dHMN: distal hereditary motor neuropathies; SS: Silver syndrome.

In the previous sections, we divided the text to better understand the specific cell situations in seipin dysfunction. However, lipodystrophy can be found in animal models with* BSCL2* knockout and in human subjects with missense or nonsense mutations. More than 30 recessive* BSCL2* loss-of-function mutations are the cause of this lipodystrophy [4, 15–31] and it is still not clear if there is significant clinical difference among these kinds of mutations. However, we do know that it is a recessive condition and that only subjects who carry mutations in both alleles manifest BSCL type 2 (Figure 7(b)), even if the mutations are in different regions of the same gene (Figure 7(c)). The lipodystrophy clinical features are out of the scope of our review and can be found elsewhere [1, 20, 104]. It is also possible to find subjects with gain-of-function mutations in seipinopathies, a dominant condition that needs at least one mutated allele to manifest the phenotypical condition (Figures 7(d) and 7(e)). To our knowledge, there is no case of a subject carrying both mutations of lipodystrophy and seipinopathy (N88S or S80L) in the same or in different alleles of the* BSCL2 *gene. The clinical features of seipinopathies are also out of the scope of our review and can be found elsewhere [5, 105–107].

Interestingly, different from seipinopathy or lipodystrophy, a rare fatal seipin neurodegenerative syndrome was also described. Referred to as Celia syndrome or progressive encephalopathy with or without lipodystrophy (PELD), this disease is caused by the R329X mutation in seipin. This is related to exon 7 gene skipping and was initially supposed to be a loss-of-function mutation. Indeed, heterozygous patients do not develop any symptoms (Figure 7(f)). However, some homozygous patients also do not develop lipodystrophy, suggesting that adipogenic functions might still be preserved (Figure 7(g)). These patients develop a fatal neurodegeneration, associated with child death by progressive encephalopathy [108–110]. On the other hand, there are some cases of compound heterozygous, when somebody inherits a lipodystrophic-related mutation and R329X mutation (Figure 7(h)). In this case, the patient has both phenotypes of progressive encephalopathy and lipodystrophy. Recently, it was shown that metreleptin intervention, used for lipodystrophy treatment, could help a compound heterozygous patient with PELD [111].

Instead of losing its function, “Celia seipin” was described as a gain-of-toxic function related to extreme ER stress. Even as a gain-of-function mutation [108–110], some heterozygous patients are completely asymptomatic (Figure 7(f)), and authors have discussed that wild-type seipin is capable of inhibiting toxic functions of “Celia seipin” [109]. Thus, this might be the rare case of gain-of-toxic-function mutation associated with recessive alleles.

## 8. Bioinformatics Predictions

**Figure 8:**
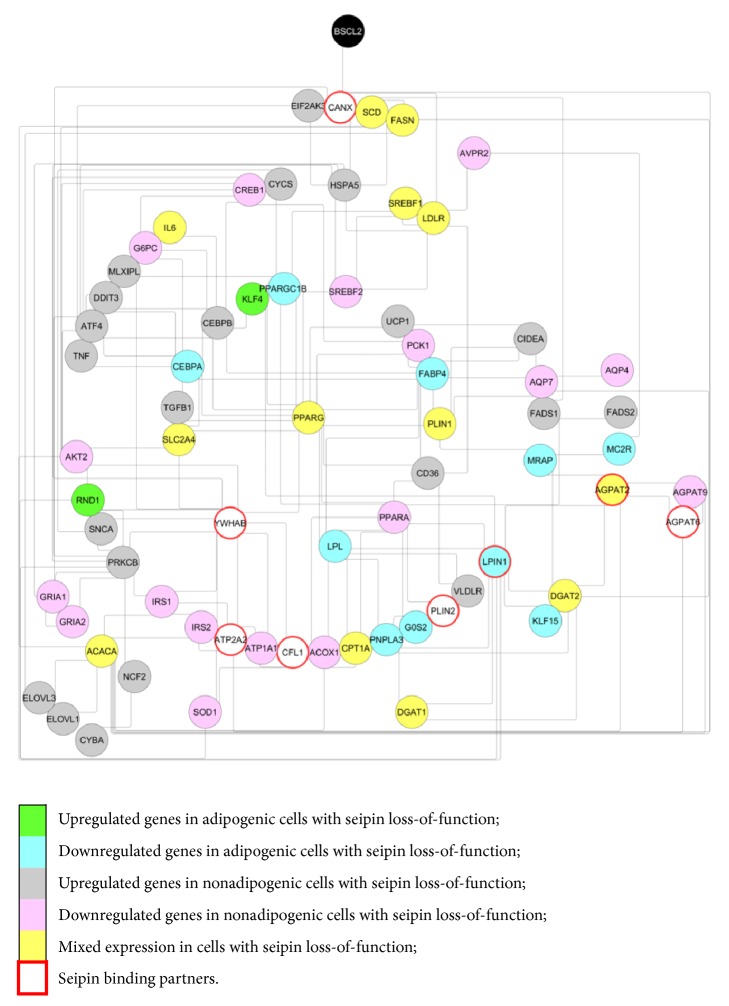
**Biological integration**. A network with genes differently regulated in seipin loss-of-function situations was created using STRING [41]. The gene list submitted is in accordance with Supplementary Tables 1 and 2. Genes painted with green are upregulated in adipogenic cells with seipin loss-of-function. Genes painted with blue are downregulated in adipogenic cells with seipin loss-of-function. Genes painted in gray are upregulated in nonadipogenic cells with seipin loss-of-function and genes painted in pink are downregulated in nonadipogenic cells with seipin loss-of-function. Genes that were observed either downregulated or upregulated in the same cell situations but in different papers are painted in yellow (mixed expression). Proteins already described as seipin physical binders are surrounded by a red circumference. The parameters chosen were “Experiments,” “Databases,” “Coexpression,” “Neighborhood,” “Gene Fusion,” “Cooccurrence,” and “Minimum required interaction score = 0.1.” Cytoscape [42] classifies the genes based on their connectivity. As the circle comes to the center, the node tends to be more connected with the network.

Aiming to integrate the knowledge about seipin, we performed some bioinformatics analysis. During the reviewing process, we collected a list of different expressed genes in loss of seipin function (Supplementary Tables 1 and 2). Therefore, we built an interactive network with all of them in Figure 8 using the STRING database [41]. The parameters chosen were “Experiments,” “Databases,” “Coexpression,” “Neighborhood,” “Gene Fusion,” “Cooccurrence,” and “Minimum required interaction score = 0.1”. It was possible to notice that seipin (referred to as* BSCL2*) has almost no relation to its own network, except for likely interacting with calnexin (referred to as* CANX*), an ER chaperone that was previously shown to bind to seipin during UPR [100]. This could mean that seipin affects gene transcription in an indirect way and UPR may have an important role in seipin loss-of-function expression genes. Cytoscape [42] was responsible for the organization of the network and as the circle comes to the center, the node tends to be more connected in the group (known as the ‘hub'). We can observe the* PPARG* gene in the center of visualization, allowing us to confirm its important role in seipin loss-of-function. As we also performed iRegulon [44], it was possible to notice that* PPARG*,* CEBPB,* and* PPARA* are transcription factors that are important to the expression of some genes. They have high enrichment scores (called “NES”): 7.278, 6.333, and 5.270, respectively.

**Figure 9:**
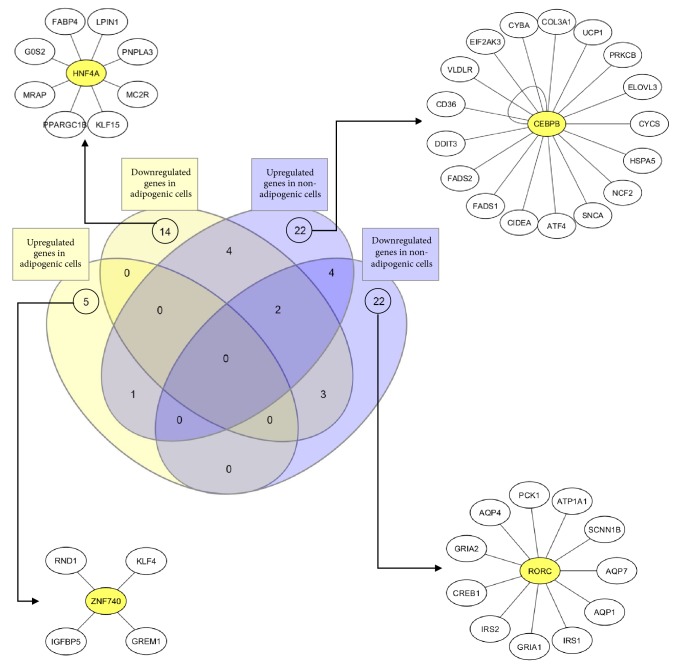
**Differently expressed genes during seipin loss-of-function**. InteractVenn [43] allowed us to see four different clusters formed when seipin loses its function in different cell types. iRegulon [44] shows* HNF4A*,* CEBPB*,* ZNF740,* and* RORC* as important transcription factors that may interfere with the expression of the observed genes for each cluster. Among them, only* CEBPB* was studied in a loss of seipin function [45].

**Figure 10:**
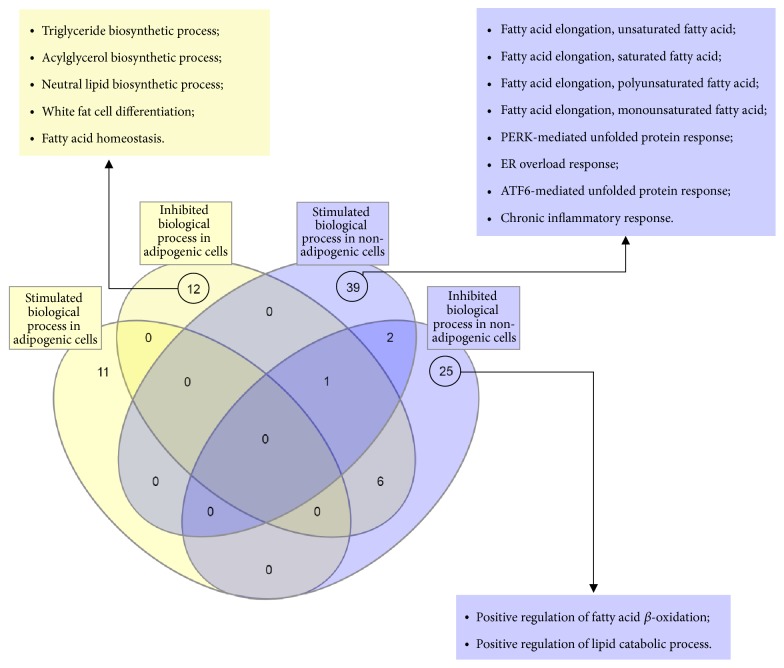
**Gene ontology for seipin loss-of-function**. InteractVenn [43] allowed us to see four different clusters formed when seipin loses its function in different cell types. All of the biological processes had a fold enrichment >100.

Figure 9 summarizes graphically the results from the grouping of the reviewed genes in Supplementary Tables 1 and 2. A Venn diagram was performed through InteractVenn [43] to allow us to observe gene expression in cells with seipin loss-of-function. It is possible to notice that the differentially expressed genes are mostly in four different clusters (circled numbers). It means that we have different gene transcription patterns in seipin loss-of-function depending on the presence of adipogenesis stimuli (yellow boxes) or not (blue boxes). Therefore, this corroborates the discussion that the consequences for the lack of seipin might be different from an adipogenic compared to a nonadipogenic cell. Of all the 77 genes reviewed (100%), only 14 (19%) are differently expressed in more than one situation. The remaining 63 (81%) are expressed in only one situation. iRegulon [44] also allowed observing important transcription factors for the expression of some genes of each one of the four clusters. Interestingly,* CEBPB* is important for the transcription of many genes upregulated in nonadipogenic cells and this gene was also already seen higher expressed in these situations [45]. Therefore, seipin may influence expression of such genes through* CEBPB* upregulation. We bring attention to* HNF4A*,* ZNF740,* and* RORC* as names for future works that might be interesting to better understand how seipin affects gene transcription.  Figure 10 also focuses on gene ontology for biological processes affected by seipin loss-of-function. We used Panther [112] to input the gene list in Supplementary Table 1 and observed what would be the processes affected by the absence of seipin function. As we have already discussed, the results point to different biological processes: in adipogenic cells, the triglyceride biosynthetic process is inhibited because of the downregulation of genes important for that process and the white fat cell differentiation is also diminished. However, in nonadipogenic cells, the positive regulation of fatty acid *β*-oxidation is also inhibited, favoring the accumulation of lipids. These are some examples that corroborate our previous discussion.

## 9. Seipin Partners

Seipin interacts physically or functionally with many proteins of ER, revealing its functions and related pathways. Nonetheless, many seipin interactions seem to be preserved in adipogenic and nonadipogenic situations, and most of the studies were performed in heterologous overexpression systems, which requires caution during the interpretation of the physiological data and extrapolation to clinical relevance. Besides, even as important findings, they still do not clearly explain the different phenotypes observed in nonadipogenic versus adipogenic cells. Thus, it is still difficult to understand the tissue-specific functions and biochemistry of seipin.

One crucial binding partner of seipin is 14-3-3*β* (UniProt gene name:* YWHAB*). This protein modulates many pathways and binds with seipin N and C terminus sequences. This phenomenon was found during 3T3-L1 mouse preadipocyte stem cell maturation, together with 14-3-3*β* and cofilin-1 interaction. The authors discussed that seipin-14-3-3*β*-cofilin-1 binding is important to actin cytoskeleton remodeling, which contributes to adipogenesis. Indeed, both 14-3-3*β* and cofilin-1 knockdown can also impair adipocyte development. However, seipin and 14-3-3*β* binding seem to be a constitutive event, also observed in nonadipogenic cells, together with 14-3-3*β* and cofilin-1 interaction [113].

Seipin was also demonstrated to bind sarco/endoplasmic reticulum Ca^2+^-ATPase (SERCA, UniProt gene name:* ATP2A2*) protein both in the* Drosophila* fat body model and in human HEK293 cells. The loss of SERCA functions is able to generate ER stress because the enzyme transports cytosolic calcium (Ca^2+^) into the ER lumen, a process that is important in adipogenesis. Seipin seems to regulate its activity and the authors discussed that the loss of* BSCL2* might prevent the increase of ER Ca^2+^ concentration and adipocyte maturation [114].

Seipin also directly interacts with adipocyte differentiation-related protein (ADRP, UniProt gene name:* PLIN2*) during adipogenesis stimulation. ADRP is important for the development and maintenance of adipose tissue. The C terminus domain of seipin is important for that binding. Yet, that interaction is not specific for adipogenic cells, but happens in HEK293 cells too. Additionally, the authors discussed how seipin loss-of-function mutations change the intracellular distribution of ADRP and how this is important in adipogenesis [67].

Gao et al. found functional interaction with a yeast perilipin protein (Pet10), which stabilizes LDs and promotes their assembly [115]. In another paper, seipin also coimmunoprecipitated with the Reep1 protein in the NIH3T3 murine model. Reep1 is necessary for adipogenesis, ER stress resistance, and ER tubular network organization [116]. However, it is still not clear how Reep1 can contribute to findings observed during seipin disorders.

Another research group also proved that seipin physically interacts with AGPAT2 and lipin1 proteins during maturation of 3T3-L1 mouse preadipocyte stem cells. These enzymes are extremely important for TG synthesis (Figure 3) and adipogenesis. The authors suggest that seipin might be required to increase the concentration of AGPAT2 and lipin1 in domains of the ER membrane. Secondly, seipin might interact with both proteins and act as a docking or scaffolding site for that complex, with a possible contribution to their activities. Seipin cytoplasmic N and C sequences showed importance to binding with lipin1, and its conserved core/looping or first transmembrane region to binding with AGPAT2 [117, 118]. Both AGPAT2 and lipin1 are important in adipocyte homeostasis, since disturbance of the first causes Berardinelli-Seip congenital lipodystrophy type 1 [119] and of the second results in* PPARG* gene downregulation [120]. However, it is important to highlight that the seipin-AGPAT2-lipin1 complex was also observed in nonadipogenic cells [117, 118]. Interestingly, Péterfy et al. observed that lipin1 is phosphorylated during adipocyte maturation and interacts with 14-3-3 proteins, which guarantee lipin1 cytoplasmic localization instead of nuclear [121]. Besides, diminution of lipin1 activity might accumulate PA, which can inhibit PPAR*γ* activity [122].

GPAT3 is one of the enzymes that catalyzes the first step of TG synthesis (Figure 3), an important process for adipose tissue development. Even with low levels in preadipocytes [123], GPAT3 mRNA transcription is upregulated during adipogenesis of 3T3-L1 mouse cells. During the process, the authors observed that GPAT3 activity was important for lipid accumulation [124], results confirmed by other groups [125]. They observed that* PPARG* is necessary to upregulate GPAT3 mRNA, increase TG accumulation, and contribute to adipogenesis [125]. Studies showed that* PPARG* mRNA silencing attenuated GPAT3 upregulation [125] and that PPAR*γ* agonists were able to increase GPAT3 mRNA transcription [123, 126]. Besides, GPAT3 mRNA silencing also attenuated adipogenesis [125]. In WAT cells, upregulation of GPAT3 was associated with TG synthesis increase and enlarged LDs [127]. Taken together, GPAT3 seems to have an important function in TG synthesis and adipogenesis. However, there is a considerable lack of information about this protein and more studies are needed to elucidate its function. Not surprisingly, GPAT3 belongs to ER, where it interacts with seipin in yeast, mammalian cells, and adipogenic and nonadipogenic tissues. Nonetheless, the authors proposed that, during adipocyte differentiation, seipin negatively regulates GPAT3 activity. They observed that increased GPAT3 activity, through its overexpression, can impair adipogenesis in seipin-deficient cells. They also observed that knocking down GPAT3 enhanced adipocyte differentiation in seipin-deficient cells [128]. The ideas presented here are not mutually exclusive, as the GPAT3 downregulation might impair adipogenesis due to diminished TG synthesis (as previously reviewed), while overactivation can activate PA, a dangerous event in adipogenesis too [128].

### 9.1. Are We All Touching Different Parts of the Same Elephant?

We would like to propose a model to understand seipin's normal function based on what Yang et al. [61] and others proposed. They showed that seipin has a conserved core/looping that is important for nonadipogenic cells. However, they also observed that seipin gained a C terminus region during evolution, which is important for adipogenesis [61]. Interestingly, the C terminus is important for binding with lipin1 and 14-3-3*β* [113, 118], while the core/looping region is fundamental to GPAT3/4 interaction [128].

**Figure 11:**
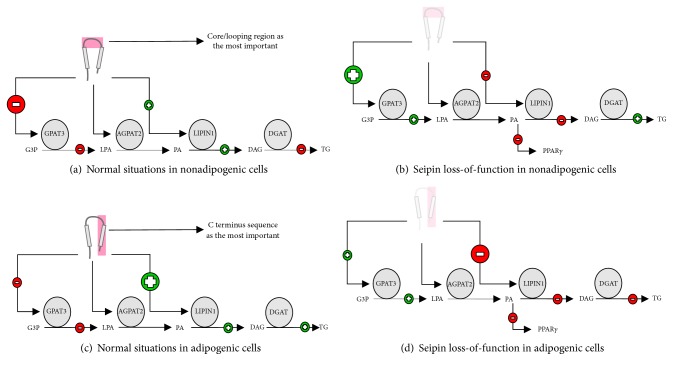
**Theoretical model for seipin**. (a) Seipin seems to have a core/looping region with more importance for nonadipogenic cells. This region can interact with GPAT3 and downregulate its activity, compromising TG synthesis. (b) Seipin loss-of-function in nonadipogenic cells seems to have the opposite behavior of the situation proposed in “(a)”. (c) Seipin also seems to have a C terminus region with more importance for adipogenesis. This region can interact with lipin1 and 14-3-3*β* to promote TG synthesis and adipogenesis. (d) Seipin loss-of-function in adipogenic cells seems to have the opposite behavior of the situation proposed in “(c)”. AGPAT: 1-acyl-sn-glycerol-3-phosphate acyltransferase; DG: diacylglycerol; DGAT: diacylglycerol o-acyltransferases; G3P: glycerol-3-phosphate; GPAT: glycerol-3-phosphate acyltransferase; LPA: lysophosphatidic acid; PA: phosphatidic acid; PPAR*γ*: peroxisome proliferator-activated receptor gamma; TG: triacylglycerol.

Here, we hypothesize that these papers are all “touching the same elephant.” This popular analogy refers to a situation in which blind men declared that they were touching a different animal when they were, in fact, all touching the same elephant. In every way, the following propositions need validation as a future perspective since our theoretical model is different from the others, because we are trying to integrate some previous publications about seipin in one basic explanation. As represented in Figure 11(a), we believe that the core/looping region of seipin is the most important in nonadipogenic cells. This sequence interacts with GPAT3/4 and negatively regulates their activities [128]. Yet, the remaining presence of the C terminus still allows the binding with lipin1 and 14-3-3*β* [113, 118]. In this way, if that C terminus domain is not the most important for these cells, lipin1 and 14-3-3*β* binding might not be enough for TG anabolism in the end. This could result in more negative stimulus to TG synthesis through more GPAT3/4 inhibition. These ideas are supported by the results of previous independent studies [61, 113, 118, 127, 128]. The main events that could happen during seipin absence caused by loss-of-function mutations are shown in Figure 11(b): TG synthesis could restart because of GPAT3/4 super stimulation [128] in nonadipogenic cells. This factor may be more important to TG synthesis than lipin1 defective-membrane association caused by the absence of seipin [117, 118]. In the same way, adipogenesis does not happen, because 14-3-3*β* does not interact with seipin in this situation to promote cytoskeleton remodeling, which contributes to adipogenesis [113]. Besides, GPAT3 super stimulation might increase PA concentration and impair PPAR*γ*, as observed by more than one study [82, 128]. PPAR*γ* seems to also protect nonadipose tissue against excessive lipid overload and maintain liver and skeletal muscle organ function, as reviewed by Kintscher and Janani [62, 129].

During adipogenesis of healthy cells (Figure 11(c)), we believe that the C terminus region of seipin might assume the control in a core/looping-almost-independent way. The C terminus sequence is not essential to GPAT3/4 and this might negatively impair the regulation of GPAT3/4 in a soft manner. However, the remaining presence of the core/looping domain still guarantees that binding. In this way, if now that the C terminus domain is the most important for these cells, lipin1 and 14-3-3*β* binding might be enough for adipogenesis and TG anabolism. This could result in more positive stimulus to TG synthesis, through intensified lipin1 and 14-3-4*β* activation, as shown by different and independent studies [61, 113, 118, 128]. During adipogenesis and seipin absence due to loss-of-function mutations (Figure 11(d)), TG synthesis can be impaired because of lipin1 activity commitment. This could increase PA production, associated with diminution of GPAT3/4 inhibition. Together, this could inhibit PPAR*γ* actions, as supported by several studies [61, 62, 128–130].

**Figure 12:**
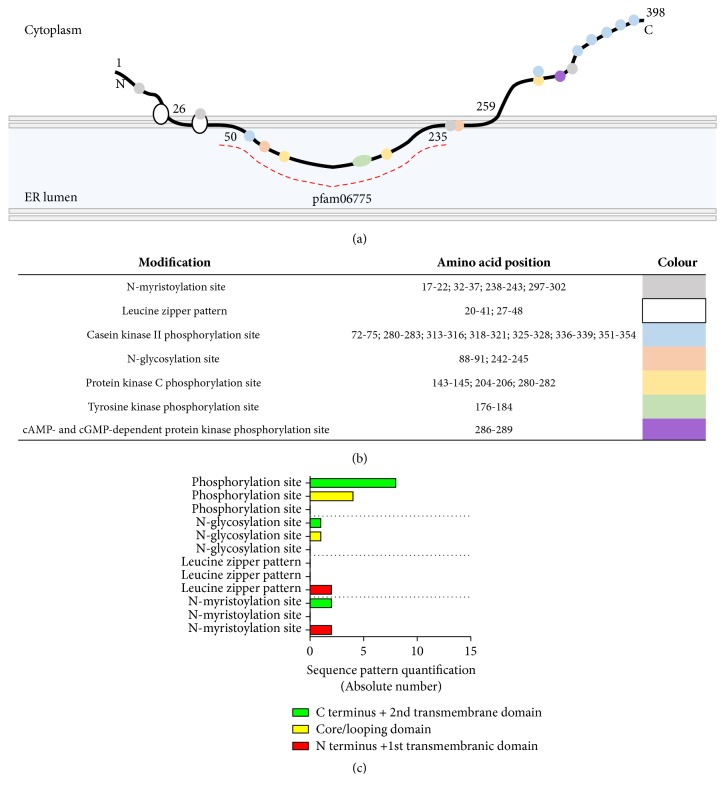
**Theoretical** **posttranslational modifications** **of seipin.** (a) Prosite [46] and Pfam [47] predictions for posttranslational modifications and conserved domains of seipin, respectively. (b) Residue patterns highlighted in (a). (c) Quantification of patterns. Even as theoretical predictions, the N-glycosylation site was already proven to occur and to be affected by the mutations N88S and S90L [48].

When we put the seipin sequence on the Prosite [46] and Pfam [47] websites, it is possible to observe the predicted posttranslational modifications and the main conserved domain: pfam06775 (Figure 12). It is true that most of these modifications were never proved to happen with seipin. However, some regions of the protein present a pattern that was recognized by system algorithms and future research can work on biochemical validation or refutation. It is possible to observe that the C terminus sequence of seipin has more phosphorylation site patterns than the core region. This may indicate that these evolutionary acquired terminus residues are regulated by the cell. Perhaps, the posttranslational modification differences between the core and C terminus sequences are responsible for the seipin function in many tissues.

This model is only theoretical and we agree with Agarwal and Garg [34] that seipin is still a mysterious protein with many biochemistry functions to be discovered and/or better characterized. Here, we tried to understand seipin proposals and suggested a unified model. Nonetheless, we are aware of the biochemistry complexity and we proposed the model to be validated or complemented in future works.

## Figures and Tables

**Table 1:** Transmembrane regions of seipin.

Isoform	Year	Transmembrane regions		Reference
		(amino acid position)		
1 (398 aa)	2001	28-49	237-258	[4]
1 (398 aa)	2004	28-49	237-258	[34]
1 (398 aa)	UniProt (accessed in 2018)	27-47	243-263	[14]
3 (462 aa)	2006	95-117	294-316	[33]
3 (462 aa)	2008	95-117	273-336	[6]

Source: reviewed papers. AA: amino acids.

## Supplementary Materials

Supplementary Materials A list of genes differently regulated in seipin altered expression situations is provided online. Supplementary Table 1 presents a list of downregulated genes in seipin loss-of-function. Supplementary Table 2 presents a list of upregulated genes in seipin loss-of-function. Supplementary Table 3 presents a list of upregulated genes in seipin gain-of-function.
