# Supplementary material for: Exploring Seipin: From Biochemistry to Bioinformatics Predictions
Source: Int J Cell Biol. 2018 Sep 19;2018:5207608. doi: 10.1155/2018/5207608 (PMC6192094; doi:10.1155/2018/5207608)
Supplement: Supplementary Materials — A list of genes differently regulated in seipin altered expression situations is provided online. Supplementary Table 1 presents a list of downregulated genes in seipin loss-of-function. Supplementary Table 2 presents a list of upregulated genes in seipin loss-of-function. Supplementary Table 3 presents a list of upregulated genes in seipin gain-of-function. [file 5207608.f1.pdf]

Hindawi
International Journal of Cell Biology
Volume 2018, Article ID 5207608
https://doi.org/10.1155/2018/5207608

Review Article

Exploring Seipin: From Biochemistry to Bioinformatics Predictions (Supplementary materials)

Aquiles Sales Craveiro Sarmento, Lázaro Batista de Azevedo Medeiros, Lucymara Fassarella Agnez-Lima, Josivan Gomes Lima and Julliane Tamara Araújo de Melo Campos.

Supplementary Table 1 - List of genes downregulated in seipin loss-of-function.

Target gene name		Seipin expression	Adipogenesis stimuli?	Cell type	Reference
From UniProt	From paper				
AATK	AATK	Seipin A212P	Yes	3T3-L1 mouse preadipocyte	[1]
ACACA	ACC1	*BSCL2* Knockout	No	Mouse Kidney Tissue	[2]
ACOX1	ACOX1	*BSCL2* Knockout	No	Mouse Kidney Tissue	[2]
AGPAT2	AGPAT2	*BSCL2* Knockdown	Yes	3T3-L1 mouse preadipocyte	[3]
AGPAT2	AGPAT2	*BSCL2* Knockdown	Yes	Mouse C3H10t1/2 cells	[4]
AKT2	AKT2	*BSCL2* Knockout	No	Mouse white adipose tissue	[5]
AKT2	AKT2	*BSCL2* Knockout	No	Mouse liver	[5]
AKT2	AKT2	*BSCL2* Knockout	No	Mouse liver	[6]
AKT2	AKT2	*BSCL2* Knockout	No	Mouse White Adipose Tissue	[6]
AKT2	AKT2	*BSCL2* Knockout	No	Mouse hepatocytes	[7]
AQP1	AQP1	*BSCL2* Knockout	No	Mouse Kidney Tissue	[2]
AQP4	AQP4	*BSCL2* Knockout	No	Mouse Kidney Tissue	[2]
AQP7	AQP7	*BSCL2* Knockout	No	Mouse Kidney Tissue	[2]
ATP1A1	ATP1A1	*BSCL2* Knockout	No	Mouse Kidney Tissue	[2]
AVPR2	V2R	*BSCL2* Knockout	No	Mouse Kidney Tissue	[2]
CEBPA	CEBPA	*BSCL2* Knockdown	Yes	3T3-L1 mouse preadipocyte	[3]
CEBPA	CEBPA	*BSCL2* Knockout	Yes	Mouse embryonic fibroblast	[8]
CEBPA	CEBPA	*BSCL2* Knockdown	Yes	Mouse C3H10t1/2 cells	[4]
CEBPA	CEBPA	*BSCL2* Knockdown	Yes	3T3-L1 mouse preadipocyte	[9]
CPT1A	CPT1A	*BSCL2* Knockout	No	Mouse hepatocytes	[7]
CPT1A	CPT1A	*BSCL2* Knockout	No	Mouse Kidney Tissue	[2]
CREB1	CREB	*BSCL2* Knockdown	No	Neurons	[10]
DGAT1	DGAT1	*BSCL2* Knockdown	Yes	3T3-L1 mouse preadipocyte	[3]
DGAT2	DGAT2	*BSCL2* Knockout	No	Mouse White Adipose Tissue	[11]
DGAT2	DGAT2	*BSCL2* Knockdown	Yes	3T3-L1 mouse preadipocyte	[3]
DGAT2	DGAT2	*BSCL2* Knockdown	Yes	Mouse C3H10t1/2 cells	[4]
FABP4	AP2	*BSCL2* Knockdown	Yes	3T3-L1 mouse preadipocyte	[3]
FABP4	AP2	*BSCL2* Knockout	Yes	Mouse embryonic fibroblast	[8]
FABP4	AP2	*BSCL2* Knockdown	Yes	Mouse C3H10t1/2 cells	[4]
FABP4	AP2	*BSCL2* Knockdown	Yes	3T3-L1 mouse preadipocyte	[9]
FASN	FAS	*BSCL2* Knockdown	No	Neurons	[10]
G0S2	G0S2	Seipin A212P	Yes	3T3-L1 mouse preadipocyte	[1]
G6PC	G6PASE	*BSCL2* Knockout	No	Primary Hepatocytes	[12]
G6PC	G6P1	*BSCL2* Knockout	No	Mouse liver	[5]
G6PC	G6P1	*BSCL2* Knockout	No	Mouse hepatocytes	[7]
GRIA1	GLUR1	*BSCL2* Knockout	No	Mouse brain	[13]
GRIA1	GLUR2	*BSCL2* Knockout	No	Mouse brain	[13]
GRIA1	GLUR1	*BSCL2* Knockdown	No	Neurons	[10]
GRIA2	GLUR2	*BSCL2* Knockdown	No	Neurons	[10]
IRS1	IRS1	*BSCL2* Knockout	No	Mouse white adipose tissue	[5]
IRS1	IRS1	*BSCL2* Knockout	No	Mouse liver	[5]
IRS1	IRS1	*BSCL2* Knockout	No	Mouse liver	[6]

Supplementary Table 1 (continuation) - List of genes downregulated in seipin loss-of-function.

Target gene name		Seipin expression	Adipogenesis stimuli?	Cell type	Reference
**From UniProt**	**From paper**				
IRS1	IRS1	*BSCL2* Knockout	No	Mouse White Adipose Tissue	[6]
IRS1	IRS1	*BSCL2* Knockout	No	Mouse hepatocytes	[7]
IRS2	IRS2	*BSCL2* Knockout	No	Mouse white adipose tissue	[5]
IRS2	IRS2	*BSCL2* Knockout	No	Mouse liver	[5]
IRS2	IRS2	*BSCL2* Knockout	No	Skeletal Muscle	[5]
IRS2	IRS2	*BSCL2* Knockout	No	Mouse liver	[6]
IRS2	IRS2	*BSCL2* Knockout	No	Mouse White Adipose Tissue	[6]
IRS2	IRS2	*BSCL2* Knockout	No	Mouse hepatocytes	[7]
KLF15	KLF15	Seipin A212P	Yes	3T3-L1 mouse preadipocyte	[1]
LDLR	LDLR	*BSCL2* Knockdown	No	Neurons	[10]
LDLR	LDLR	*BSCL2* Knockdown	Yes	3T3-L1 mouse preadipocyte	[9]
LPIN1	LIPIN1	*BSCL2* Knockdown	Yes	3T3-L1 mouse preadipocyte	[3]
LPIN1	LIPIN1	*BSCL2* Knockdown	Yes	Mouse C3H10t1/2 cells	[4]
LPL	LPL	*BSCL2* Knockdown	Yes	Mouse C3H10t1/2 cells	[4]
MC2R	MC2R	Seipin A212P	Yes	3T3-L1 mouse preadipocyte	[1]
MGAT1	MGAT1	*BSCL2* Knockdown	Yes	3T3-L1 mouse preadipocyte	[3]
MRAP	MRAP	Seipin A212P	Yes	3T3-L1 mouse preadipocyte	[1]
MTTP	MTP	*BSCL2* Knockout	No	Mouse hepatocytes	[7]
MTTP	MTP	*BSCL2* Knockout	No	Mouse liver	[5]
MTTP	MTP	*BSCL2* Knockout	No	Mouse liver	[6]
PCK1	PEPCK1	*BSCL2* Knockout	No	Mouse liver	[5]
PDZK1	PDZK1	Seipin A212P	Yes	3T3-L1 mouse preadipocyte	[1]
PLIN1	PLIN1	*BSCL2* Knockout	Yes	Mouse embryonic fibroblast	[8]
PLIN1	PERILIPIN A	*BSCL2* Knockdown	Yes	3T3-L1 mouse preadipocyte	[9]
PNPLA3	ADIPONUTRIN	*BSCL2* Knockdown	Yes	3T3-L1 mouse preadipocyte	[3]
PPARA	PPARA	*BSCL2* Knockout	No	Mouse liver	[6]
PPARA	PPARA	*BSCL2* Knockout	No	Mouse Kidney Tissue	[2]
PPARA	PPARA	*BSCL2* Knockout	No	HepG2 cells	[14]
PPARA	PPARA	*BSCL2* Knockout	No	Mouse hepatocytes	[7]
PPARG	PPARG	*BSCL2* Knockout	No	Mouse brain	[15].
PPARG	PPARG	*BSCL2* Knockdown	Yes	3T3-L1 mouse preadipocyte	[3]
PPARG	PPARG	*BSCL2* Knockout	Yes	Mouse embryonic fibroblast	[8]
PPARG	PPARG	*BSCL2* Knockdown	Yes	Mouse C3H10t1/2 cells	[4]
PPARG	PPARG	*BSCL2* Knockout	No	Mice neurons	[16]
PPARG	PPARG	*BSCL2* Knockdown	Yes	3T3-L1 mouse preadipocyte	[9]
PPARGC1B	PPARGC1B	Seipin A212P	Yes	3T3-L1 mouse preadipocyte	[1]
PREF-1	PREF1	*BSCL2* Knockout	No	Mouse White Adipose Tissue	[8]
SCD	SCD	*BSCL2* Knockdown	No	Neurons	[10]
SCNN1B	SCNN1B	*BSCL2* Knockout	No	Mouse Kidney Tissue	[2]
SOD1	SOD1	*BSCL2* Knockout	No	Mouse Kidney Tissue	[2]
SLC12A2	NCC	*BSCL2* Knockout	No	Mouse Kidney Tissue	[2]
SLC12A3	NCC	*BSCL2* Knockout	No	Mouse Kidney Tissue	[2]

Supplementary Table 1 (continuation) - List of genes downregulated in seipin loss-of-function.

Target gene name		Seipin expression	Adipogenesis stimuli?	Cell type	Reference
From UniProt	From paper				
SLC2A4	GLUT4	*BSCL2* Knockout	No	Mouse White Adipose Tissue	[5]
SLC2A4	GLUT4	*BSCL2* Knockout	No	Mouse White Adipose Tissue	[6]
SLC2A4	GLUT4	*BSCL2* Knockdown	Yes	Mouse C3H10t1/2 cells	[4]
SLC2A4	GLUT4	*BSCL2* Knockdown	Yes	3T3-L1 mouse preadipocyte	[9]
SLC2A4	GLUT4	*BSCL2* Knockout	No	Mouse Kidney Tissue	[2]
SREBF1	SREBP1C	*BSCL2* Knockdown	No	Neurons	[10]
SREBF1	SREBP1C	*BSCL2* Knockdown	Yes	3T3-L1 mouse preadipocyte	[3]
SREBF1	SREBP1C	*BSCL2* Knockdown	Yes	Mouse C3H10t1/2 cells	[4]
SREBF2	SREBP2	*BSCL2* Knockdown	No	Neurons	[10]
THRSP	THRSP	Seipin A212P	Yes	3T3-L1 mouse preadipocyte	[1]
TMCC3	TMCC3	Seipin A212P	Yes	3T3-L1 mouse preadipocyte	[1]

Supplementary Table 2 - List of genes upregulated in seipin loss-of-function.

Target gene name		Seipin expression	Adipogenesis stimuli?	Cell type	Reference
**From UniProt**	**From paper**				
ACACA	ACC	*BSCL2* Knockout	No	HepG2 cells	[14]
AGPAT2	AGPAT2	*BSCL2* Knockout	No	Mouse White Adipose Tissue	[11]
AGPAT2	AGPAT2	*BSCL2* Knockout	No	HepG2 cells	[14]
ATF4	ATF4	*BSCL2* Knockout	No	HepG2 cells	[14]
CD36	CD36	*BSCL2* Knockout	No	HepG2 cells	[14]
CEBPB	CEBPB	*BSCL2* Knockout	No	Mouse White Adipose Tissue	[8]
CIDEA	CIDEA	*BSCL2* Knockout	No	Mouse White Adipose Tissue	[8]
CIDEA	CIDEA	*BSCL2* Knockout	No	HepG2 cells	[14]
COL3A1	COL3A1	*BSCL2* Knockout	No	Mouse Kidney Tissue	[2]
CPT1A	CPT1	*BSCL2* Knockout	No	Mouse White Adipose Tissue	[8]
CYBA	P22PHOX	*BSCL2* Knockout	No	Mouse Kidney Tissue	[2]
CYCS	CYTC	*BSCL2* Knockout	No	Mouse White Adipose Tissue	[8]
DDIT3	CHOP	*BSCL2* Knockout	No	HepG2 cells	[14]
DGAT1	DGAT1	*BSCL2* Knockout	No	Mouse hepatocytes	[7]
EIF2AK3	PERK	*BSCL2* Knockout	No	HepG2 cells	[14]
ELOVL1	ELOVL1	*BSCL2* Knockout	No	Mouse White Adipose Tissue	[11]
ELOVL3	ELOVL3	*BSCL2* Knockout	No	Mouse White Adipose Tissue	[8]
ELOVL3	ELOVL3	*BSCL2* Knockout	No	Mouse White Adipose Tissue	[11]
FADS1	FADS1	*BSCL2* Knockout	No	Mouse White Adipose Tissue	[11]
FADS2	FADS2	*BSCL2* Knockout	No	Mouse White Adipose Tissue	[11]
FASN	FAS	*BSCL2* Knockout	No	Mouse liver	[5]
FASN	FAS	*BSCL2* Knockout	No	HepG2 cells	[14]
FASN	FAS	*BSCL2* Knockout	No	Mouse hepatocytes	[7]
FASN	FASN	*BSCL2* Knockout	No	Primary Hepatocytes	[12]
GREM1	GREM1	Seipin A212P	Yes	3T3-L1 mouse preadipocyte	[1]
HSPA5	GRP78	*BSCL2* Knockout	No	HepG2 cells	[14]
IGFBP5	IGFBP5	Seipin A212P	Yes	3T3-L1 mouse preadipocyte	[1]
IL6	IL6	*BSCL2* Knockout	No	Mice Hippocampus	[17]
IL6	IL6	Seipin A212P	Yes	3T3-L1 mouse preadipocyte	[1]
IL6	IL6	*BSCL2* Knockout	No	Mice neurons	[16]
KLF4	KLF4	Seipin A212P	Yes	3T3-L1 mouse preadipocyte	[1]
MGAT1	MGAT1	*BSCL2* Knockout	No	Primary Hepatocytes	[12]
MLXIPL	CHREBP	*BSCL2* Knockout	No	Mouse hepatocytes	[7]
MMP13	MMP13	Seipin A212P	Yes	3T3-L1 mouse preadipocyte	[1]
NCF2	P67PHOX	*BSCL2* Knockout	No	Mouse Kidney Tissue	[2]
PLIN1	PLIN	*BSCL2* Knockout	No	HepG2 cells	[14]
PPARG	PPARG	*BSCL2* Knockout	No	Mouse liver	[5]
PPARG	PPARG	*BSCL2* Knockout	No	Mouse liver	[6]
PPARG	PPARG	*BSCL2* Knockout	No	HepG2 cells	[14]
PRKCB	PKCB	*BSCL2* Knockout	No	Mouse Kidney Tissue	[2]
RND1	RND1	Seipin A212P	Yes	3T3-L1 mouse preadipocyte	[1]

Supplementary Table 2 (continuation) - List of genes upregulated in seipin loss-of-function.

Target gene name		Seipin expression	Adipogenesis stimuli?	Cell type	Reference
From UniProt	From paper				
SCD	SCD1	*BSCL2* Knockout	No	Primary Hepatocytes	[12]
SCD	SCD1	*BSCL2* Knockout	No	Mouse liver	[5]
SCD	SCD1	*BSCL2* Knockout	No	Mouse liver	[6]
SCD	SCD1	*BSCL2* Knockout	No	Mouse liver	[14]
SCD	SCD1	*BSCL2* Knockout	No	Mouse hepatocytes	[7]
SNCA	αSYN	*BSCL2* Knockout	No	Mice neurons	[16]
SREBF1	SCREBP1	*BSCL2* Knockout	No	HepG2 cells	[14]
TGFB1	TGFB	*BSCL2* Knockout	No	Mouse Kidney Tissue	[2]
TNF	TNF	*BSCL2* Knockout	No	Mice Hippocampus	[17]
UCP1	UCP1	*BSCL2* Knockout	No	Mouse White Adipose Tissue	[8]
VLDLR	VLDLR	*BSCL2* Knockout	No	Mouse White Adipose Tissue	[11]

Supplementary Table 3 - List of genes upregulated in seipin gain-of-function.

Target gene name		Seipin expression	Adipogenesis stimuli?	Cell type	Reference
**From UniProt**	**From paper**				
DDIT3	CHOP	Seipin N88S	No	Mouse brain neuroblastoma (N2a)	[18]
DDIT3	CHOP	Seipin N88S	No	Mouse brain neuroblastoma (N2a)	[19]
DDIT3	CHOP	Seipin S90L	No	Mouse brain neuroblastoma (N2a)	[19]
HERPUD1	HERP	Seipin N88S	No	Mouse brain neuroblastoma (N2a)	[19]
HERPUD1	HERP	Seipin S90L	No	Mouse brain neuroblastoma (N2a)	[19]
HSP90B1	GRP94	Seipin N88S	No	Mouse brain neuroblastoma (N2a)	[19]
HSP90B1	GRP94	Seipin S90L	No	Mouse brain neuroblastoma (N2a)	[19]
HSPA5	BIP	Seipin N88S	No	Mouse brain neuroblastoma (N2a)	[18]
HSPA5	BIP	Seipin S90L	No	Mouse brain neuroblastoma (N2a)	[19]
HSPA5	BIP	Seipin N88S	No	Mouse brain	[20]
P4HB	PDI	Seipin N88S	No	Mouse brain	[20]
STC2	STC2	Seipin N88S	No	Mouse brain neuroblastoma (N2a)	[19]
STC2	STC2	Seipin S90L	No	Mouse brain neuroblastoma (N2a)	[19]

References

1. Qiu W, Wee K, Takeda K, Lim X, Sugii S, Radda GK, et al. Suppression of Adipogenesis by Pathogenic Seipin Mutant Is Associated with Inflammatory Response. PLoS One. 2013;8. doi:10.1371/journal.pone.0057874

2. Liu X-J, Wu X-Y, Wang H, Wang S-X, Kong W, Zhang L, et al. Renal injury in Seipin-deficient lipodystrophic mice and its reversal by adipose tissue transplantation or leptin administration alone: adipose tissue–kidney crosstalk. FASEB J. 2018;32: 1–13. doi:10.1096/fj.201701427R

3. Chen W, Yechoor VK, Chang BH-J, Li M V., March KL, Chan L. The human lipodystrophy gene product Berardinelli-Seip congenital lipodystrophy 2/seipin plays a key role in adipocyte differentiation. Endocrinology. 2009;150: 4552–4561. doi:10.1210/en.2009-0236

4. Payne VA, Grimsey N, Tuthill A, Virtue S, Gray SL, Nora ED, et al. The human lipodystrophy gene BSCL2/Seipin may be essential for normal adipocyte differentiation. Diabetes. 2008;57: 2055–2060. doi:10.2337/db08-0184

5. Cui X, Wang Y, Tang Y, Liu Y, Zhao L, Deng J, et al. Seipin ablation in mice results in severe generalized lipodystrophy. Hum Mol Genet. 2011;20: 3022–3030. doi:10.1093/hmg/ddr205

6. Gao M, Wang M, Guo X, Qiu X, Liu L, Liao J, et al. Expression of seipin in adipose tissue rescues lipodystrophy, hepatic steatosis and insulin resistance in seipin null mice. Biochem Biophys Res Commun. 2015;460: 143–150. doi:10.1016/j.bbrc.2015.02.147

7. Xu P, Wang H, Kayoumu A, Wang M, Huang W, Liu G. Diet rich in Docosahexaenoic Acid/Eicosapentaenoic Acid robustly ameliorates hepatic steatosis and insulin resistance in seipin deficient lipodystrophy mice. Nutr Metab (Lond). 2015;12: 10. doi:10.1186/s12986-015-0054-x

8. Chen W, Chang B, Saha P, Hartig SM, Li L, Reddy VT, et al. Berardinelli-Seip Congenital Lipodystrophy 2/Seipin Is a Cell-Autonomous Regulator of Lipolysis Essential for Adipocyte Differentiation. Mol Cell Biol. 2012;32: 1099–1111. doi:10.1128/MCB.06465-11

9. Yang W, Thein S, Guo X, Xu F, Venkatesh B, Sugii S, et al. Seipin differentially regulates lipogenesis and adipogenesis through a conserved core sequence and an evolutionarily acquired C-terminus. Biochem J. 2013;452: 37–44. doi:10.1042/BJ20121870

10. Wei S, Soh SLY, Qiu W, Yang W, Seah CJY, Guo J, et al. Seipin regulates excitatory synaptic transmission in cortical neurons. J Neurochem. 2013;124: 478–489. doi:10.1111/jnc.12099

11. Chen W, Zhou H, Liu S, Fhaner CJ, Gross BC, Lydic TA, et al. Altered lipid metabolism in residual white adipose tissues of Bscl2 deficient mice. PLoS One. 2013;8. doi:10.1371/journal.pone.0082526

12. Chen W, Zhou H, Saha P, Li L, Chan L. Molecular mechanisms underlying fasting modulated liver insulin sensitivity and metabolism in male lipodystrophic Bscl2/seipin-deficient mice. Endocrinology. 2014;155: 4215–4225. doi:10.1210/en.2014-1292

13. Zhou L, Chen T, Li G, Wu C, Wang C, Li L, et al. Activation of PPARγ Ameliorates Spatial Cognitive Deficits

through Restoring Expression of AMPA Receptors in Seipin Knock-Out Mice. J Neurosci. 2016;36: 1242–1253. doi:10.1523/JNEUROSCI.3280-15.2016

14. Lounis MA, Lalonde S, Rial SA, Bergeron KF, Ralston JC, Mutch DM, et al. Hepatic BSCL2 (Seipin) Deficiency Disrupts Lipid Droplet Homeostasis and Increases Lipid Metabolism via SCD1 Activity. Lipids. 2017;52: 129–150. doi:10.1007/s11745-016-4210-5

15. Zhou L, Yin J, Wang C, Liao J, Liu G, Chen L. Lack of seipin in neurons results in anxiety-and depression-like behaviors via down regulation of PPARG. Hum Mol Genet. 2014;23: 4094–4102. doi:10.1093/hmg/ddu126

16. Wang L, Hong J, Wu Y, Liu G, Yu W, Chen L. Seipin deficiency in mice causes loss of dopaminergic neurons via aggregation and phosphorylation of α-synuclein and neuroinflammation. Cell Death Dis. 2018;9: 13. doi:10.1038/s41419-018-0471-7

17. Qian Y, Yin J, Hong J, Li G, Zhang B, Liu G, et al. Neuronal seipin knockout facilitates Aβ-induced neuroinflammation and neurotoxicity via reduction of PPARγ in hippocampus of mouse. J Neuroinflammation. Journal of Neuroinflammation; 2016;13: 145. doi:10.1186/s12974-016-0598-3

18. Ito D, Fujisawa T, Iida H, Suzuki N. Characterization of seipin/BSCL2, a protein associated with spastic paraplegia 17. Neurobiol Dis. 2008;31: 266–277. doi:10.1016/j.nbd.2008.05.004

19. Ito D, Suzuki N. Molecular pathogenesis of Seipin/BSCL2-related motor neuron diseases. Ann Neurol. 2007;61: 237–250. doi:10.1002/ana.21070

20. Yagi T, Ito D, Nihei Y, Ishihara T, Suzuki N. N88S seipin mutant transgenic mice develop features of seipinopathy/BSCL2-related motor neuron disease via endoplasmic reticulum stress. Hum Mol Genet. 2011;20: 3831–3840. doi:10.1093/hmg/ddr304
